# Effects of foliar nitrogen application on morphological and physiological characteristics in newly emerged leaves of *Bambusa emeiensis*

**DOI:** 10.3389/fpls.2026.1751417

**Published:** 2026-03-30

**Authors:** Jiangyu Feng, Dan Wang, Xingyu Wang, Bing Yu, Lixia Yu, Huijin Fan, Lushuang Li, Hui Zhan, Shuguang Wang, Lingfeng Li

**Affiliations:** 1 Biological Research and Utilization Innovation Team in Bamboo Resources of Yunnan Province, Southwest Forestry University, Kunming, China; 2 Key Laboratory of Forest Resources Conservation and Utilization in the Southwest Mountains of China, Ministry of Education, Southwest Forestry University, Kunming, China

**Keywords:** *Bambusa emeiensis*, foliar nitrogen, leaf morphology, photosynthetic pigments, sugar metabolism, antioxidant enzyme activities

## Abstract

**Introduction:**

Bamboo growth is highly nitrogen-dependent, particularly during the branch and leaf extension phase. Foliar nitrogen application offers a precise and efficient alternative to soil fertilization. However, little is known about its effects on the growth and physiology of developing leaves in bamboos.

**Methods:**

In this study, different concentrations of urea (1%, 2%, 3%, 4%, and 5%) were applied to the newly emerged leaves of *Bambusa emeiensis*. Leaf samples were collected at 7, 14, and 21 days after treatment. The effects of foliar urea application on morphological development, sugar metabolism, and oxidative stress responses were assessed.

**Results:**

The foliar application of 0.3% urea significantly enhanced leaf development, as evidenced by increased leaf length, width, and thickness. It also boosted the content of photosynthetic pigments and the activities of sugar-metabolizing enzymes, leading to greater photoassimilate accumulation. Concurrently, it elevated the activities of antioxidant enzymes (superoxide dismutase, catalase, and peroxidase), which reduced the levels of hydrogen peroxide and malondialdehyde.

**Discussion:**

These findings indicate that moderate foliar urea application enhances leaf development and stress tolerance in *B. emeiensis* by promoting photoassimilate production and antioxidant capacity. This provides a physiological basis for precision nutrient management in bamboo cultivation.

## 1 Introduction

Bamboo, belonging to the Poaceae family and the Bambusoideae subfamily, is an important component of forest ecosystems. It possesses significant ecological functions and boasts a wide range of applications, including construction materials, bridges, bamboo crafts, pulp production, biofuels, etc (Zhou et al., 2005). It’s widely distributed in southwestern China, *Bambusa emeiensis* is a major source for bamboo weaving and papermaking owing to its favorable characteristics, including long internodes, thin culm walls, and long, tough fibers (Liu, 2022; Qi et al., 2013; Tan et al., 2020; Xie et al., 2017). After a bamboo shoot emerges above ground, it can complete its vertical growth within 35–40 days (Guo et al., 2023). As one of the fastest-growing bamboo species, *B. emeiensis* requires substantial nutrients during its growth and development stage (Lin et al., 2021; Piouceau et al., 2014; Tiziani et al., 2023).

Fertilization, as a crucial agricultural management practice, effectively supplements essential nutrients for plant growth and development, ultimately determining yield and quality (Panhwar et al., 2019; Cao et al., 2024; Liu et al., 2023; Li et al., 2021a; Fan et al., 2025). Nitrogen is a macronutrient indispensable for plant growth and development, playing key roles in multiple physiological processes (Zhang et al., 2024). These processes primarily drive leaf growth by regulating mesophyll cell division and expansion while enhancing chlorophyll biosynthesis (Ding et al., 2025; Luo et al., 2024; Tian et al., 2022a; Xie et al., 2025). Studies have shown that under greenhouse conditions, nitrogen application in one-year-old *Dendrocalamus asper* significantly increased its biomass accumulation (Januarie and Kadyampakeni, 2025). In *Phyllostachys edulis* forests, nitrogen fertilization promoted bamboo shoot growth and modified the lignification process (Yang et al., 2022). Similarly, nitrogen application in *Dendrocalamopsis oldhami* induced significant changes in the leaf morphology (Yin et al., 2023). Fertilization primarily refers to either root or foliar methods, differing in the pathway of nutrient delivery to the plant (Niu et al., 2021). Root fertilization often leads to soil acidification, compaction, and environmental pollution. In contrast, foliar fertilization has garnered increasing research attention due to its rapid nutrient uptake, high efficiency, and lower environmental risk (Ding et al., 2023). Urea (CH^4^N^2^O) is the preferred nitrogen source for foliar fertilization due to its rapid uptake and translocation (Li et al., 2021b; Wang et al., 2024). Studies across species, including mandarin, maize, and cowpea, confirm that foliar urea can reduce fertilizer input while enhancing yield, quality, and stress resistance (Dong et al., 2023; Oleiwi and Al-Falahy, 2023; Zhao et al., 2020; Senthamil et al., 2025). However, the regulatory role of foliar nitrogen in bamboo growth and physiology remains unclear.

The branch and leaf extension phase represents the key growth stage in bamboo development. During this period, bamboo exhibits optimum capacity for nitrogen uptake, driven by high root activity and sink demand in expanding tissues, which can effectively reduce the nitrogen fertilizer losses through volatilization, runoff, and leaching (He et al., 2024; Jin et al., 2010; Wu et al., 2024). Increased nitrogen application at this stage promotes branch and leaf growth, which enhances photosynthetic efficiency and provides energy for dry matter accumulation. Furthermore, rational nitrogen application during the branch and leaf extension phase enhances plant stress resistance capacity (Gou et al., 2017). However, most existing studies focus on soil nitrogen application, leaving the efficacy and physiological impact of foliar nitrogen during this critical underexplored (Cao et al., 2024; He et al., 2024; Januarie and Kadyampakeni, 2025; Liu et al., 2023; Tan et al., 2020) Therefore, whether foliar nitrogen application optimizes leaf growth and stress resilience in bamboo at this stage remains to be tested.

This study was conducted on *B. emeiensis* to investigate the effects of foliar urea application during the branch and leaf extension phase. Newly emerged leaves were administered varying concentrations of urea solution, and key traits were assessed to elucidate the regulatory role of foliar nitrogen in leaf growth and stress resistance. For bamboos like *B. emeiensis* grown on slopes, foliar fertilization offers a strategic advantage by enabling direct canopy nutrition, bypassing soil-related issues like fixation and leaching. This supports precision forestry and facilitates scalable drone-assisted spraying, reducing both labor and environmental costs. Therefore, this study not only advances the theoretical framework for nitrogen management in bamboo, but also provides physiological evidence to support these innovative, sustainable cultivation practices.

## 2 Materials and methods

### 2.1 Plant materials and fertilizer treatment

**Figure 1:**
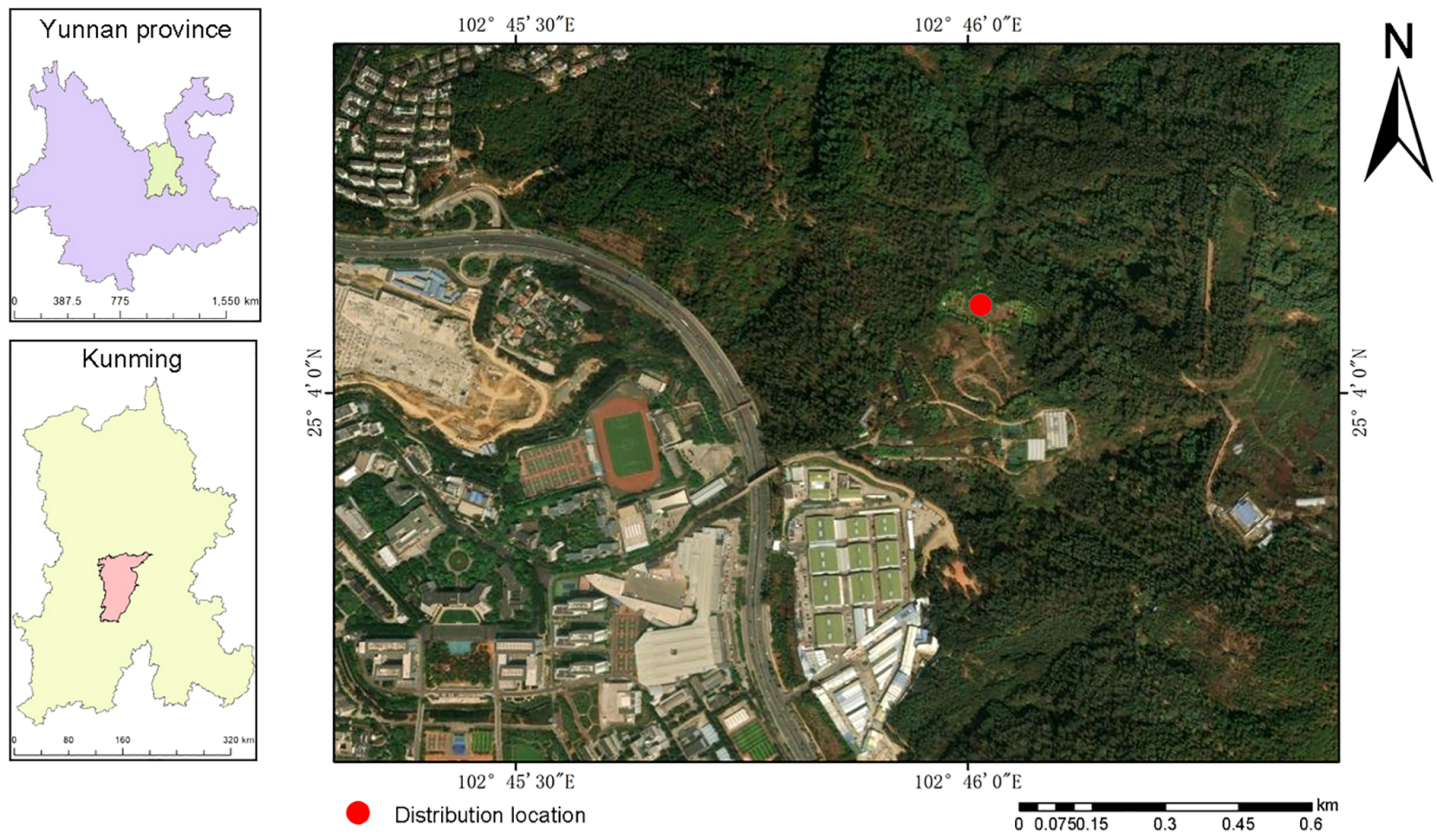
Geographical location of the bamboo garden behind the mountain of Southwest Forestry University.

The experimental plant material, *B. emeiensis, was* cultivated in the bamboo garden of Southwest Forestry University, Kunming, Yunnan Province, China (102°46′7″E, 25°3′53″N) (**Figure 1**), at an altitude of 1,936 meters. This site is characterized by a northern low-latitude subtropical plateau mountain monsoon climate, with a mean annual temperature of approximately 16.5 °C and precipitation of about 1450 mm. The soil at the experimental site was classified as red soil, and its basic physicochemical properties are presented in **Table 1**.

**Table 1:** Physicochemical properties of soils in the bamboo garden.

pH	Organic matter (%)	Total nitrogen (%)	Total phosphorus (mg kg^-^¹)	Total potassium (g kg^-^¹)
7.10	0.81	0.07	573.67	2.67

In May 2024, newly emerged young bamboo, free of diseases and pests and exhibiting uniform growth vigor, were selected from two-year-old bamboo clumps as experimental materials. All selected bamboo plants grew under the same soil conditions and were at the branch and leaf expansion stage, with a leaf length of 8.91 ± 1.03 cm and a leaf width of 1.89 ± 0.17 cm, approximately half the size of mature leaves. The control group was sprayed with distilled water. For the experimental groups, urea concentrations (0.1%, 0.2%, 0.3%, 0.4%, and 0.5%) were determined based on established protocols (Vaddi et al., 2025; Jespersen and Huang, 2015) and a preliminary test which indicated that concentrations at or above 1.0% caused phytotoxicity (leaf scorching) in our bamboo species. The foliar application was carried out at 16:00 under clear, windless, and rain-free conditions. The recorded meteorological conditions for the month were as follows: temperature, 19.8 °C; relative humidity, 65%; precipitation, 51 mm; and sunshine duration, 135.6 hours. Three spray applications were administered at three-day intervals over nine days to five selected plants per treatment using a handheld sprayer. The fertilizer solution was ensured to be uniformly applied to both the upper and lower leaf surfaces. A total volume of 400 mL was applied per plant. Fresh leaves were randomly collected from the upper, middle, and lower layers of the canopy. Sampling began on day 7 after fertilization and was repeated weekly, for a total of three sampling events. Sampling commenced on day 7 after fertilization, with fresh leaves randomly collected from the upper, middle, and lower layers of the canopy. This process was repeated weekly for a total of three sampling events. The bamboo species used in this study was identified as *Bambusa emeiensis* L.C. Chia & H.L. Fung by Prof. Shuguang Wang (Biological Research and Utilization Innovation Team in Bamboo Resources of Yunnan Province, Southwest Forestry University, Kunming, China). Although no new voucher specimen was deposited for the present study, species identification was verified by comparison with an authenticated herbarium specimen (KUN No. 1015362) deposited at the Herbarium of Kunming Institute of Botany, Chinese Academy of Sciences (Herbarium code: KUN) (**Supplementary Figure 1**).

### 2.2 DNA barcoding analysis

Genomic DNA was extracted from fresh leaf tissue using a commercial plant DNA extraction kit according to the manufacturer’s instructions. The chloroplast trnL (UAA) intron region was amplified using the universal primers c and f described by Taberlet et al. (1991). The primer sequences were trnL-c (5′-CGAAATCGGTAGACGCTACG-3′) and trnL-f (5′-ATTTGAACTGGTGACACGAG-3′). PCR amplification was performed in a thermal cycler with an initial denaturation at 94 °C for 1 min, followed by 5 cycles of 94 °C for 30 s, 50 °C for 40 s, and 72 °C for 1 min, then 35 cycles of 94 °C for 30 s, 54 °C for 40 s, and 72 °C for 1 min, with a final extension at 72 °C for 10 min and a hold at 4 °C. PCR products were examined by capillary electrophoresis using an ABI 3130XL Genetic Analyzer (Applied Biosystems, USA). Amplified fragments were purified and sequenced bidirectionally by a commercial sequencing service. Chromatograms were manually inspected to ensure sequence quality, and the forward and reverse reads were assembled to obtain a consensus sequence.

Positive and negative controls were included in all PCR reactions to confirm amplification specificity and to exclude contamination. The resulting trnL (UAA) intron sequence of *B. emeiensis* generated in this study has been deposited in the GenBank database under accession number PZ094212. The obtained sequence was compared with sequences available in the NCBI nucleotide database using BLASTn for species identification. The top BLAST hits (20 sequences) of the trnL region were retrieved from the NCBI database. Multiple sequence alignment was performed using MEGA software, and a phylogenetic tree was constructed using the Neighbor-Joining method with 1000 bootstrap replicates to further verify species identity.

### 2.3 Determination of soil basic chemical properties

Soil pH was measured using a pH meter (Ministry of Ecology and Environment of the People’s Republic of China, 2018). Soil organic matter was quantified by the volumetric method (State Bureau of Technical Supervision of the People’s Republic of China, 1988). Total nitrogen content was analyzed using an elemental analyzer (State Forestry Administration of the People’s Republic of China, 2015a). Total phosphorus was assessed by spectrophotometry (Ministry of Environmental Protection of the People’s Republic of China, 2011), while total potassium was measured using atomic absorption spectrophotometry (State Forestry Administration of the People’s Republic of China, 2015b).

### 2.4 Measurement of leaf length and width

For each treatment, ninety newly emerged leaves were randomly selected, and their leaf length and width were measured following the protocol described by Zhao et al. (2025) using a measuring ruler.

### 2.5 Anatomical structure of plant leaves

Twenty fresh leaves were randomly selected from plant in each treatment and fixed in the FAA solution (90% ethanol + 5% glacial acetic acid + 5% formaldehyde). The samples underwent ethanol gradient dehydration, paraffin embedding, and sectioning using a microtome (Leica RM2165, Germany) to obtain 7 μm-thick sections. The starch localization assay was performed as described previously (Chu, 1963). Briefly, sections were treated with a 0.5% potassium periodate solution for 10 min and then stained with Schiff’s reagent for 30 min. Following dehydration through a graded ethanol series, the sections were counterstained with Fast Green FCF. The slides were sealed using Canada balsam (Solarbio, China) and observed under an optical microscope (PH100-3B41L-IPL, Phenix, China) equipped with Image View software (DS-3000, Caikang, China).

### 2.6 Determination of moisture content

Leaf moisture content (LMC) analysis was performed as previously described (Li and He, 2021). Ninety-newly emerged leaves were randomly selected per treatment. Their fresh weight was determined using an analytical balance. The leaves were then oven-dried at 80 °C until a constant weight was achieved. The leaf water content was calculated using the following equation:


Water Content (%)=[(Fresh weight−Dry weight)/Fresh weight]×100

### 2.7 Determination of photosynthetic and enzymatic activities

A total of 350 fresh leaves were randomly selected from each treatment, ground into fine powder using a grinder.

#### 2.7.1 Determination of photosynthetic pigments

For pigment extraction, a 0.1 g sample was weighed and homogenized with quartz sand and calcium carbonate, followed by extraction with 95% ethanol. Pigment content was determined by UV-spectrophotometer through absorbance measurements at 470, 649, and 665 nm (Lichtenthaler, 1987). The concentrations were calculated using the following equations:


$$C_{a}\left(µg/mL\right)=13.95A_{665}–6.88A_{649}$$

$$C_{b}\left(µg/mL\right)=24.96A_{649}–7.32A_{665}$$

$$C_{total}\;\left(µg/mL\right)=C_{a}+Cb$$

$$C_{car}\;\left(µg/mL\right)=\left(1000A_{470}–2.05C_{a}–114.8C_{b}\right)/245$$

The pigment content was expressed on a fresh weight basis as milligrams per gram of sample (mg/g). It was calculated using the following equation: [C (µg/mL) × V (mL) × D]/[W (g) × 1000], where C is the pigment concentration, V is the extract volume, D is the dilution factor, and W is the sample fresh weight.

#### 2.7.2 Determination of soluble sugars and starch contents

The phenol-sulfuric acid method was employed to determine the soluble sugar (Okahisa et al., 2006) and starch content (Glassop et al., 2007). The non-structural carbohydrate (NSC) was calculated as the sum of soluble sugars and starch.

#### 2.7.3 Assay of enzymatic activities related to sugar and starch metabolism

A 0.1 g sample was weighed and homogenized, followed by centrifugation at 6900 × g for 10 minutes. The supernatant was collected for subsequent analysis. Activities of soluble acid invertase (SAI) and sucrose synthase (SuSy) were determined spectrophotometrically, with assays conducted according to established protocols (Moscatello et al., 2011; Sung et al., 1994; Wang et al., 2019a). The pellet from the above step was collected and used for the determination of cell wall acid invertase (CWI) activity following the method of Wang et al. (2019a).

The activities of sucrose phosphate synthase (SPS), ADP-glucose pyrophosphorylase (AGPase), granule-bound starch synthase (GBSS), and soluble starch synthase (SSS) were determined according to previously described methods (Hubbard et al., 1989; Nakamura et al., 1989). Starch phosphorylase (STP) activity was assayed using the method described by Appeldoorn et al. (1997). The activities of α-amylase and β-amylase were determined following the method of Liu (2007). Enzyme activity was expressed as μmol NADH per gram fresh weight per hr (μmol NADH·g^-^¹ FW·h^-^¹).

#### 2.7.4 Determination of malondialdehyde and hydrogen peroxide (H^2^O^2^) content

MDA content was determined using the thiobarbituric acid (TBA) method, as described by Cai (2013). Specifically, 0.2 g of leaf sample was homogenized in 10 mL of pre-cooled phosphate buffer (pH 7.8) on ice and then centrifuged at 4 °C for 15 min. A 1.5 mL aliquot of the supernatant was transferred to a stoppered test tube, mixed with 2.5 mL of 0.5% TBA solution, and incubated in a boiling water bath for 20 min. After cooling, the mixture was centrifuged, and the absorbance of the supernatant was measured at 532, 600, and 450 nm. The MDA content was expressed as μmol per gram fresh weight (μmol·g^-^¹ FW) calculated using the following formula:


$$\text{MDA}\left(\mu mol/L\right)=6.45\left({\text{OD}}_{532}-{\text{OD}}_{600}\right)-0.56{\text{OD}}_{450}$$

H^2^O^2^ content was measured using a commercial assay kit (Suzhou Michy Biomedical Technology Co., Ltd, China) according to the manufacturer’s instructions.

#### 2.7.5 Assay of superoxide dismutase, catalase, and peroxidase activities

The crude enzyme extract was prepared by homogenizing 0.2 g of leaf tissue with 10 mL of pre-cooled phosphate buffer (pH 7.8) on ice, followed by centrifugation at 4 °C for 15 min. SOD activity was analyzed by the nitroblue tetrazolium (NBT) photochemical reduction method (Chen and Wang, 2006). CAT activity was determined using the ultraviolet absorption assay (Li, 2024). Peroxidase POD activity was measured according to the guaiacol method (Gao, 2006).

### 2.8 Data analysis

The experimental data represent mean values from nine replicates, consisting of three biological replicates (*n* = 3) each with three technical replicates. Statistical analyses were performed using SPSS 27.0 software, including one-way analysis of variance (ANOVA) and independent samples t-tests. The Duncan test was employed for *post-hoc* analysis, with differences considered statistically significant at *p* < 0.05. Figures were generated using Origin software, and data tables were prepared using Excel.

## 3 Result

### 3.1 DNA barcoding

The chloroplast trnL–trnF region was successfully amplified and sequenced from sample. Sequence chromatograms showed clear peaks without ambiguous base calls, and both positive and negative controls yielded expected results (**Supplementary Figure 3**). BLASTn analysis against the NCBI nucleotide database indicated that the obtained sequence showed 100% identity with several *Bambusa* species, including *B. emeiensis*, *Bambusa tulda*, and *Bambusa cornigera* (query coverage: 100%, E-value: 0.0) (**Supplementary Figure 4**). Considering that the trnL–trnF region is a relatively conserved chloroplast marker and may have limited resolution among closely related *Bambusa* species, the molecular results, together with morphological characteristics, support the identification of the studied plant material as *B. emeiensis*.

### 3.2 Effects of nitrogen fertilization on the morphology and water content of newly emerged leaves

**Figure 2:**
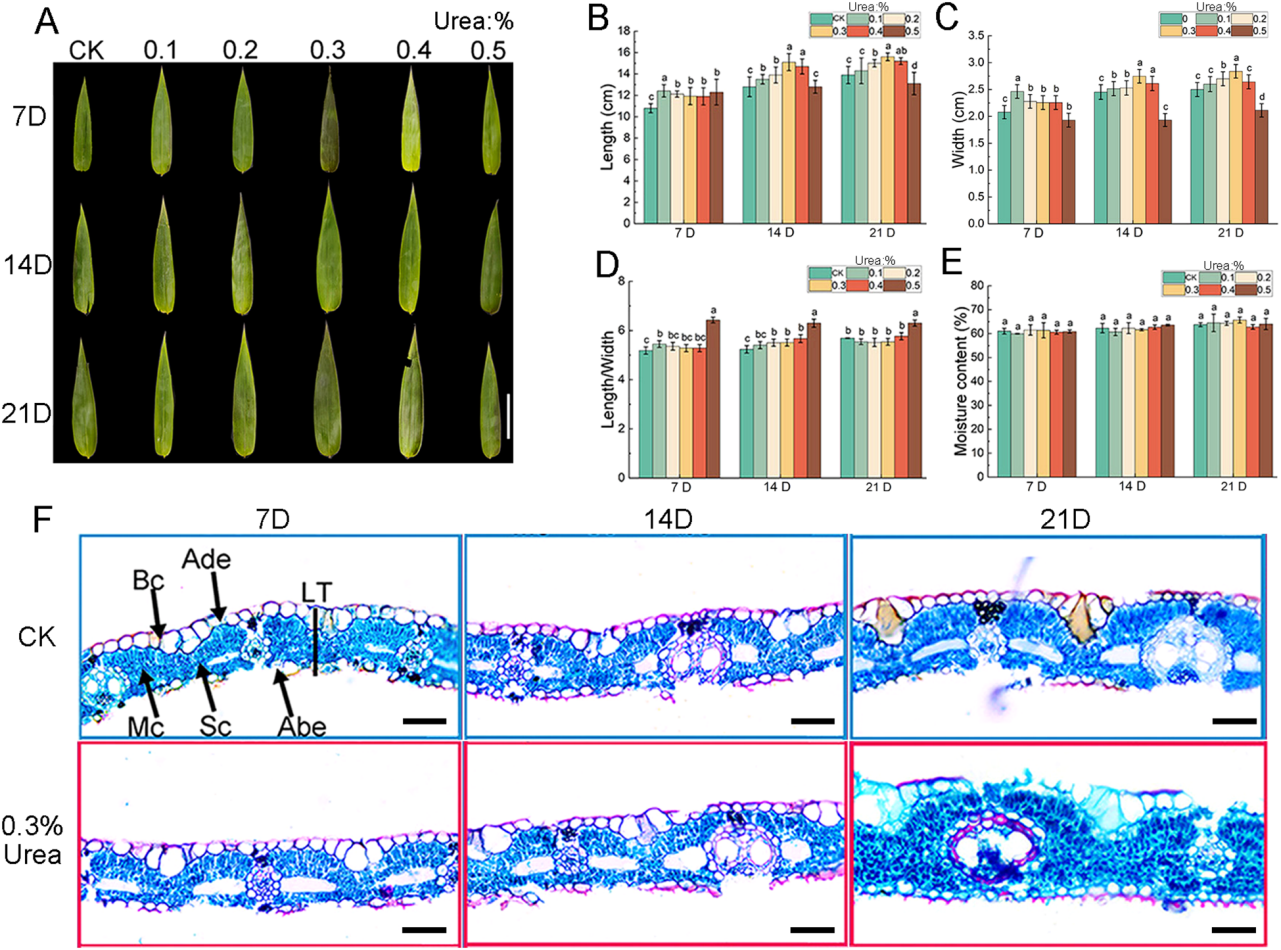
Effects of urea (CH^4^N^2^O) foliar spraying on leaf morphology and water content of *Bambusa emeiensis*. **(A)** Leaf phenotypic images of the control and treatment groups (Scale: 5 cm), **(B)** Leaf length, **(C)** leaf width, **(D)** length-to-width ratio, **(E)** moisture content. **(F)** Application of 0.3% urea on the anatomical structure of newly developed leaves (Scale: 50 μm). Blue and red boxes indicate the control and fertilizer-treated groups, respectively. Leaves were sampled at 7, 14, and 21 days after spraying under water (control) and 0.3% urea conditions, respectively. Ade, adaxial epidermis; Abe, abaxial epidermis; Mc, mesophyll cell; Bc, bulliform cell; Sc, fusiform cell; LT, leaf thickness. 7 DAY, 14 DAY, and 21 DAY denote the 7th, 14th, and 21st day after fertilization, respectively. Data are presented as means ± SD (*n* = 3, combining three biological replicates with three technical replicates each). Different letters with the same growth period indicated significant differences at *p* < 0.05 according to the Duncan’s test.

Leaf morphology serves as a comprehensive representation of plant growth status and environmental adaptability. To evaluate the effects of nitrogen fertilization on leaf morphology in *B. emeiensis*, the developing leaves of young bamboos (**Supplementary Figure 2**) were treated with urea solutions at different concentrations. Most treatment groups exhibited an increasing trend in leaf length and width compared to the control, with the 0.3% urea treatment resulting in the maximum values for both traits. This indicates that low-concentration urea promoted leaf growth and development (**Figures 2A–C**). Moreover, leaves treated with 0.5% urea showed significantly reduced length and width compared to the control, while exhibiting a notably higher length-to-width ratio than other treatments (**Figures 2A–D**), suggesting that high urea concentration (0.5%) inhibits leaf expansion, with a more pronounced effect on leaf width. These findings collectively indicated that appropriate nitrogen application may enhance the photosynthetic capacity of *B. emeiensis* by facilitating leaf area expansion, thereby laying the foundation for biomass accumulation, plant growth and development.

Leaf water content is a critical parameter in plant physiological and ecological research, serving as a comprehensive indicator of plant water status, physiological functionality, and environmental adaptability. The water content of newly emerged leaves exhibited an overall increasing trend throughout leaf development, reaching its maximum during the 21^st^ day of foliar application (**Figure 2E**), suggesting that the leaves were undergoing an active stage of morphogenesis. At the 21^st^ day after treatment with 0.3% urea, leaf water content showed the increment of 3.04% compared to control (**Figure 2E**). The application of nitrogen fertilizer promoted plant vegetative growth, leading to rapid leaf cell expansion. This process was accompanied by water uptake to maintain turgor pressure, consequently resulting in elevated leaf water content (Amoah et al., 2025; He et al., 2024; Kim et al., 2018). These results suggest that the foliar nitrogen application may optimize the plant’s water management strategy, thereby enhancing leaf growth vigor, photosynthetic capacity, and stress resistance efficiency.

### 3.3 Effects of foliar application on anatomical structure of *Bambusa emeiensis* leaves

Nitrogen nutrition could effectively influence the plant cell morphology (Zhang et al., 2017). The variations in leaf morphology following nitrogen application, particularly with the 0.3% urea treatment, suggested underlying alterations in mesophyll cells. Transverse sections of leaves revealed a significant increase in leaf thickness during 21-days after spraying with 0.3% urea (**Supplementary Table 1**; **Figure 2F**), which may enhance leaf mechanical strength and thereby reduce the probability of leaf damage. This increase in leaf thickness was manifested at the cellular level as increased thickness of upper epidermal and mesophyll cells (**Supplementary Table 1**; **Figure 2F**). Furthermore, the increased thickness of upper epidermal cells is often accompanied by cuticle thickening, which helps reduce leaf water loss. We also assessed the size of fusoid cells and bulliform cells, finding that their cross-sectional area expanded markedly after the 0.3% urea treatment (**Supplementary Table 1**; **Figure 2F**). This demonstrates that the nitrogen application effectively promotes the expansion of these cells, potentially enhancing light capture and distribution capacity within leaves. In summary, foliar nitrogen application to young *B. emeiensis* leaves promoted the expansion of epidermal cells, mesophyll cells, bulliform cells, and fusiform cells. These structural modifications at the cellular level may collectively improve photosynthetic efficiency and strengthen their adaptive capabilities.

### 3.4 Influence of foliar application on photosynthetic pigments and assimilates in newly emerged plant leaves

**Figure 3:**
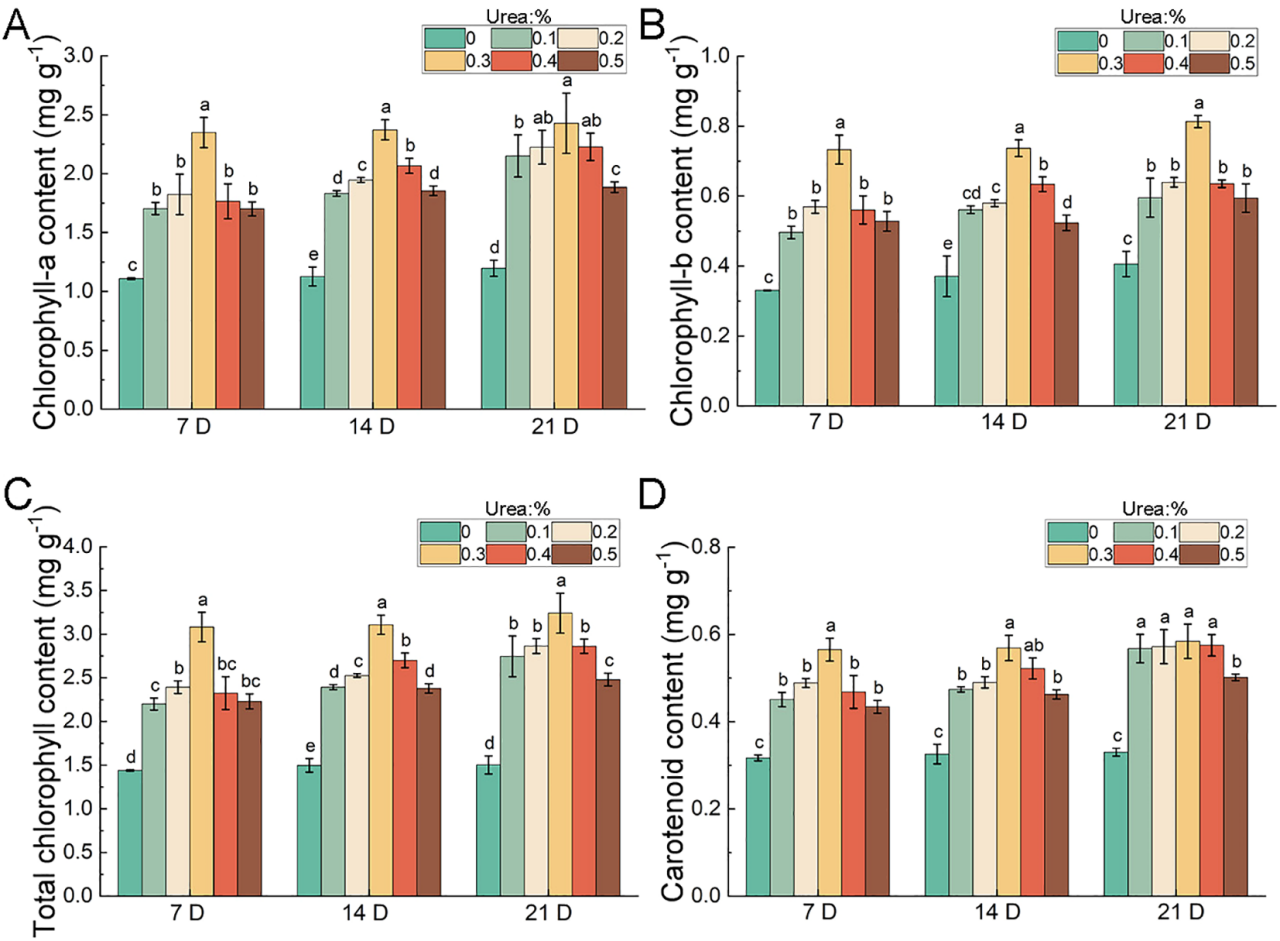
Effects of urea (CH^4^N^2^O) foliar spraying on photosynthetic pigments in leaves of *B. emeiensis*. **(A)** Chlorophyll a, **(B)** Chlorophyll b, **(C)** Total chlorophyll, and **(D)** Carotenoid content. 7 DAY, 14 DAY, and 21 DAY denote the 7th, 14th, and 21st day after fertilization, respectively. Different letters with the same growth period indicated significant differences at *p* < 0.05 according to the Duncan’s test.

Photosynthetic pigments are vital secondary metabolites in plants. Their content not only determines photosynthetic efficiency but also indirectly reflects leaf nutritional status, responses to environmental stress, and growth stages (Cechin et al., 2022; Jiang et al., 2022). Compared to the control, all treatment groups exhibited upregulated photosynthetic pigment, with the 0.3% urea treatment consistently showing the optimum levels (**Figures 3A–D**). Chlorophyll a and b reached their peak concentrations at 21-days after the 0.3% urea treatment, respectively. Urea application also enhanced leaf carotenoid content, following a similar pattern to chlorophyll, with the 0.3% urea treatment yielding the highest carotenoid levels. Furthermore, the window from the 14^th^ to 21^st^ day after urea application emerged as the critical phase for carotenoid accumulation (**Figure 3D**). These results indicated that the nitrogen fertilization enhanced photosynthetic capacity in bamboo leaves by increasing chlorophyll content. At the same time, the concurrent rise in carotenoids provides photoprotection, thereby maintaining high photosynthetic efficiency under high-light stress condition.

**Figure 4:**
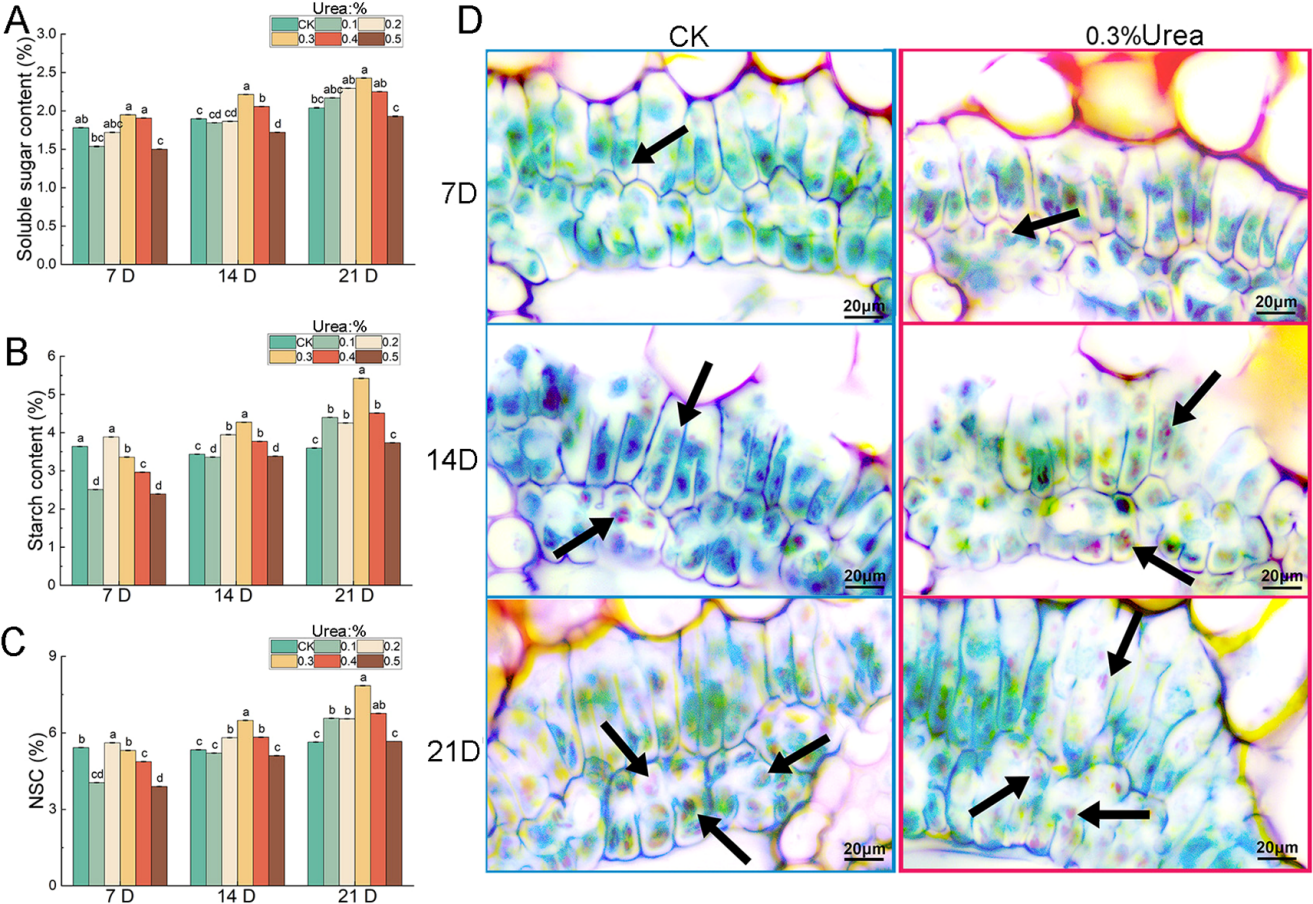
Effects of urea foliar spraying on assimilates in leaves of *B. emeiensis*. **(A)** Soluble sugar content, **(B)** Starch content, **(C)** Non-structural carbohydrate (NSC) content, **(D)** Localization of starch granules in leaves of *B. emeiensis*. Blue and red boxes indicate the control and fertilizer-treated groups, respectively. Leaves at 7, 14, and 21 days after water and 0.3% urea spraying, respectively. The black arrow indicates the starch grains. 7 DAY, 14 DAY, and 21 DAY denote the 7th, 14th, and 21st day after fertilization, respectively. Different letters with the same growth period indicated significant differences at *p* < 0.05 according to the Duncan’s test.

Photosynthetic assimilates are primarily stored as soluble sugars and starch, which provide the essential energy and structural basis for plant growth and development (Sun et al., 2022; Wang et al., 2023). The leaf content of NSC, comprising soluble sugars and starch was determined. The results showed an increasing trend in soluble sugar and starch content was monitored at 21-days after different fertilizer treatments compared to the control (**Figures 4A,B**). Consistently, the 0.3% urea treatment resulted in the highest increment in soluble sugars and starch, which rose by 18.97% and 50.74%, respectively (**Figures 4A–C**). Given that 0.3% urea most significantly increased the starch content in leaves, we subsequently examined starch distribution in leaf sections treated with this concentration. Compared with the control group, the accumulation of starch granules within mesophyll cells exhibited a gradual increase trend (**Figure 4D**). This indicated that foliar nitrogen application can effectively promote the accumulation of photosynthetic assimilates, providing energy support for plant growth and stress resistance. Due to its most pronounced effects on leaf growth and photosynthetic assimilate accumulation, leaves treated with 0.3% urea were chosen for all subsequent analyses.

### 3.5 Effects of nitrogen fertilization on key enzyme activities in sucrose and starch metabolism

**Figure 5:**
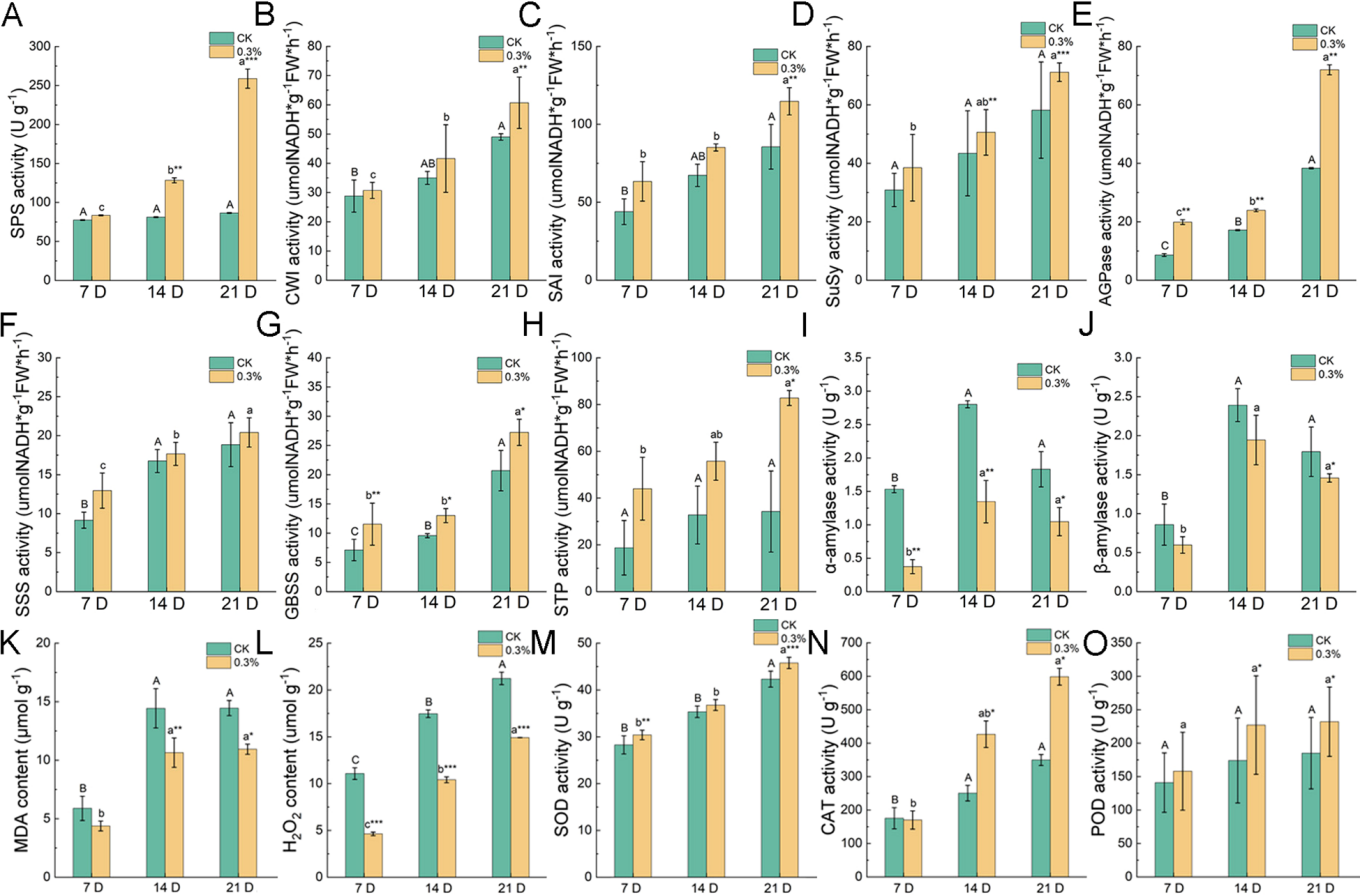
Effects of foliar spraying with 0.3% urea on sucrose metabolism-related enzyme activities and antioxidant system in leaves of *B. emeiensis*. **(A)** SPS, **(B)** CWI, **(C)** SAI, **(D)** SuSy, **(E)** AGPase, **(F)** SSS, **(G)** GBSS, **(H)** STP, **(I)** α-amylase, **(J)** β-amylase, **(K)** MDA content, **(L)** H^2^O^2^ content, **(M)** SOD activity, **(N)** CAT activity, and **(O)** POD activity. 7 DAY, 14 DAY, and 21 DAY denote the 7th, 14th, and 21st day after fertilization, respectively. Upper case letters indicate significant differences among sampling days for the control group, while lower case letters denote significant differences among sampling days for the treatment group. *P*-values were determined using Student’s t-test for comparisons between the treatment and control groups. *P < 0.05, **P < 0.01.***P < 0.001.

The metabolism of soluble sugars and starch is closely regulated by key enzymes including SAI, CWI, SuSy, and SPS, all of which play vital roles in plant growth, development, and stress responses (Liu et al., 2018; Verma et al., 2017, Verma et al., 2019). After treatment with 0.3% urea, the activities of SAI, CWI, SuSy, and SPS in leaves were consistently higher than those in the control at all measured time points (**Figures 5A–D**). The upregulation of SPS activity, a key enzyme in sucrose synthesis, suggested that the accumulation of soluble sugars might be attributed to enhanced sucrose biosynthesis. However, the concurrent increase in the activities of sucrose-degrading enzymes, SuSy, CWI, and SAI, implied that sucrose was being decomposed into monosaccharides to support leaf development.

AGPase, GBSS, SSS, STP, α-amylase, and β-amylase plays key roles in starch synthesis and degradation (Fan et al., 2024a; Liang et al., 2022; Zhang et al., 2016). We first analyzed the activities of starch-synthesizing enzymes, including AGPase, GBSS, SSS, and STP, and found that their activities gradually increased during leaf development (**Figures 5E–H**). Moreover, the activities of these enzymes were higher in the 0.3% urea treatment group compared to the control (**Figures 5E–H**), indicating an enhancement in the enzymatic direction of starch synthesis, which aligned with the previously observed increase in the starch content in leaves (**Figure 4D**). In contrast, the activities of starch-degrading enzymes, such as α-amylase and β-amylase, were lower in the 0.3% urea treatment than control (**Figures 5I, J**). In summary, nitrogen fertilization increased the activities of starch-synthesizing enzymes while decreasing those of starch-degrading enzymes in bamboo leaves, leading to starch accumulation and providing an energy reserve for subsequent growth.

### 3.6 Effects of oxidative stress in *Bambusa emeiensis* leaves during foliar application

MDA and H^2^O^2^ are key oxidative metabolites that accumulate in plants under stress conditions, playing a dual role in plant physiology as markers of oxidative damage and participants in signal transduction and defense responses (Morshedloo et al., 2025; Sarwar et al., 2025). MDA content revealed a steady and significant increase during leaf development, optimum at 14-days, and stabilized by 21^st^-day after N treatment (**Figure 5K**). Compared to the control, the leaves treated with 0.3% urea showed reduced MDA content (**Figure 5K**). The H^2^O^2^ content also exhibited an upward trend during leaf development, but significantly lower in the 0.3% urea treatment compared to the control (**Figure 5L**). These results indicated that the foliar urea application might reduce oxidative stress levels, enhanced cell membrane stability, and mitigated oxidative damage during leaf development.

The activities of three key antioxidant enzymes (SOD, CAT, and POD) were also determined in the leaves (**Figures 5M–O**). The results showed a constantly increasing trend in SOD, CAT, and POD activities during leaf development, with significantly higher activities in the 0.3% urea treatment group compared to the control (**Figures 5M–O**). In conclusion, the nitrogen application effectively enhanced the environmental adaptability and stress resistance capacity of *B. emeiensis* by maintaining redox homeostasis and reducing membrane lipid peroxidation damage in leaves.

### 3.7 Correlation analysis

**Figure 6:**
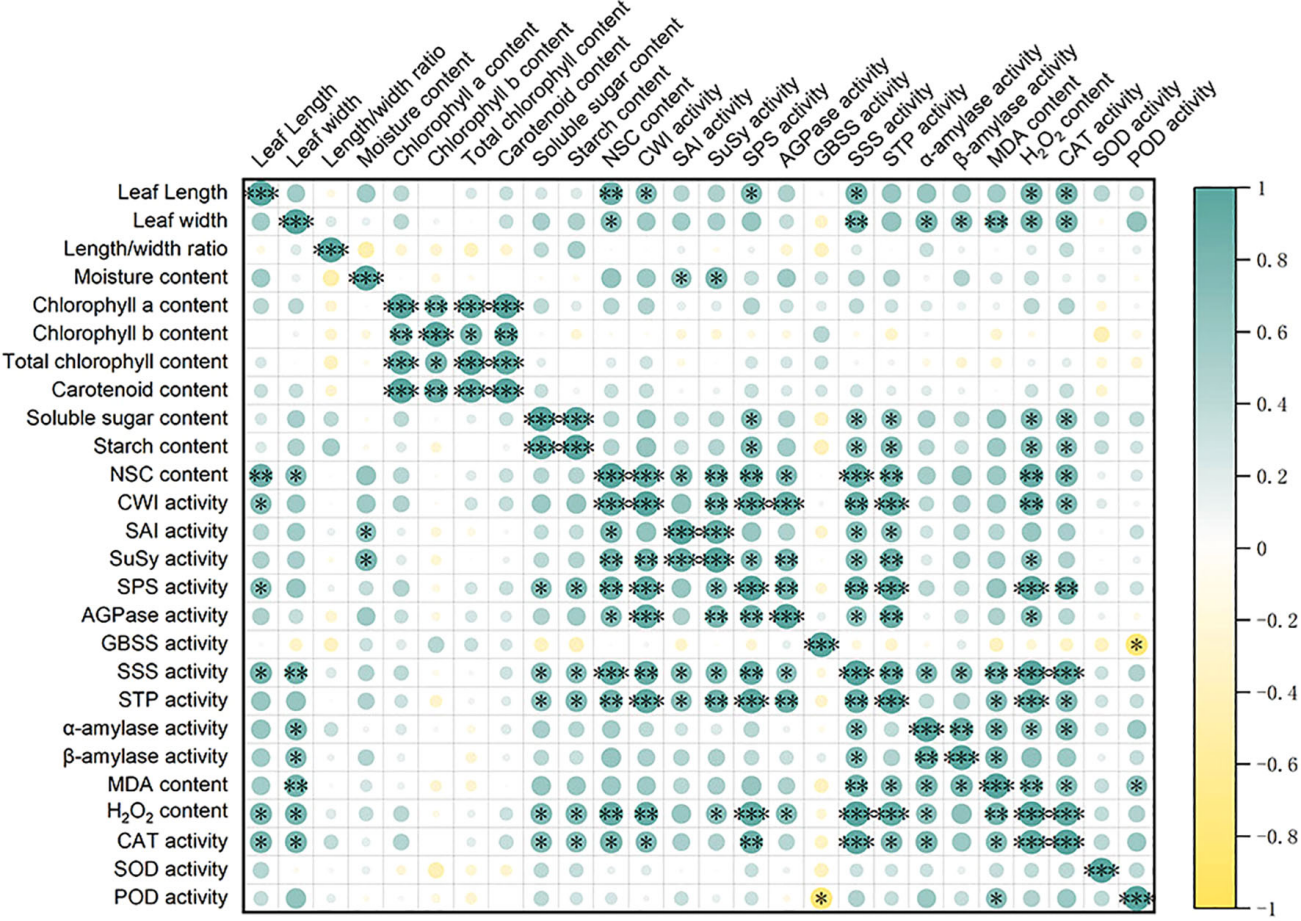
Correlation analysis of leaf physiological parameters in *B. emeiensis* in response to foliar spraying of 0.3% urea. The matrix displays Pearson correlation coefficients. A green-to-yellow color gradient represents the strength and direction of correlation, with green indicating positive values and yellow indicating negative values. The size and color intensity of the circles are proportional to the absolute value of the correlation coefficient. *P*-values were determined using Student’s t-test for comparisons between the treatment and control groups. *P < 0.05, **P < 0.01.***P < 0.001.

To further elucidate the impact of foliar urea application on the morphology and physiology of new leaves in *B. emeiensis*, a correlation analysis was performed on a suite of leaf physiological indices (**Figure 6**). Significant positive correlations were observed among the contents of various photosynthetic pigments. This is consistent with the data presented in **Figures 3A–D**, which shows that the pigment concentrations upregulated significantly with increasing nitrogen application rates and reached saturation at the rate of 0.3%. This aligned with the fundamental physiological role of nitrogen as a key constituent in the synthesis of chlorophyll and other pigments, suggesting that nitrogen supplementation promoted the integrated development of the photosynthetic apparatus.

Furthermore, the contents of NSC showed widespread positive correlations with the activities of key sucrose-metabolizing enzymes (SuSy, SPS, SAI, CWI) and starch-synthesizing enzymes (AGPase, SSS). This complex network of relationships indicated that the foliar urea application activated the synthesis and turnover of photoassimilates. The enhanced activities of synthesizing enzymes directly facilitated the accumulation of photosynthetic products. In contrast, the concomitant increase in catabolic enzyme activities likely supplied ample carbon skeletons and energy to support vigorous growth and development.

Additionally, a positive correlation was observed between the levels of H^2^O^2^ and MDA, and the activities of CAT and POD enzymes. Although this correlation suggests that the accumulation of H^2^O^2^ and MDA can induce a higher antioxidant enzyme activity, the fertilizer treatment ultimately resulted in lower levels of H^2^O^2^ and MDA compared to the control. This indicates that nitrogen fertilization enhanced the antioxidant enzyme system, which effectively scavenged reactive oxygen species (ROS) and thereby alleviated membrane lipid peroxidation and related damage.

### 3.8 Principal component analysis

**Figure 7:**
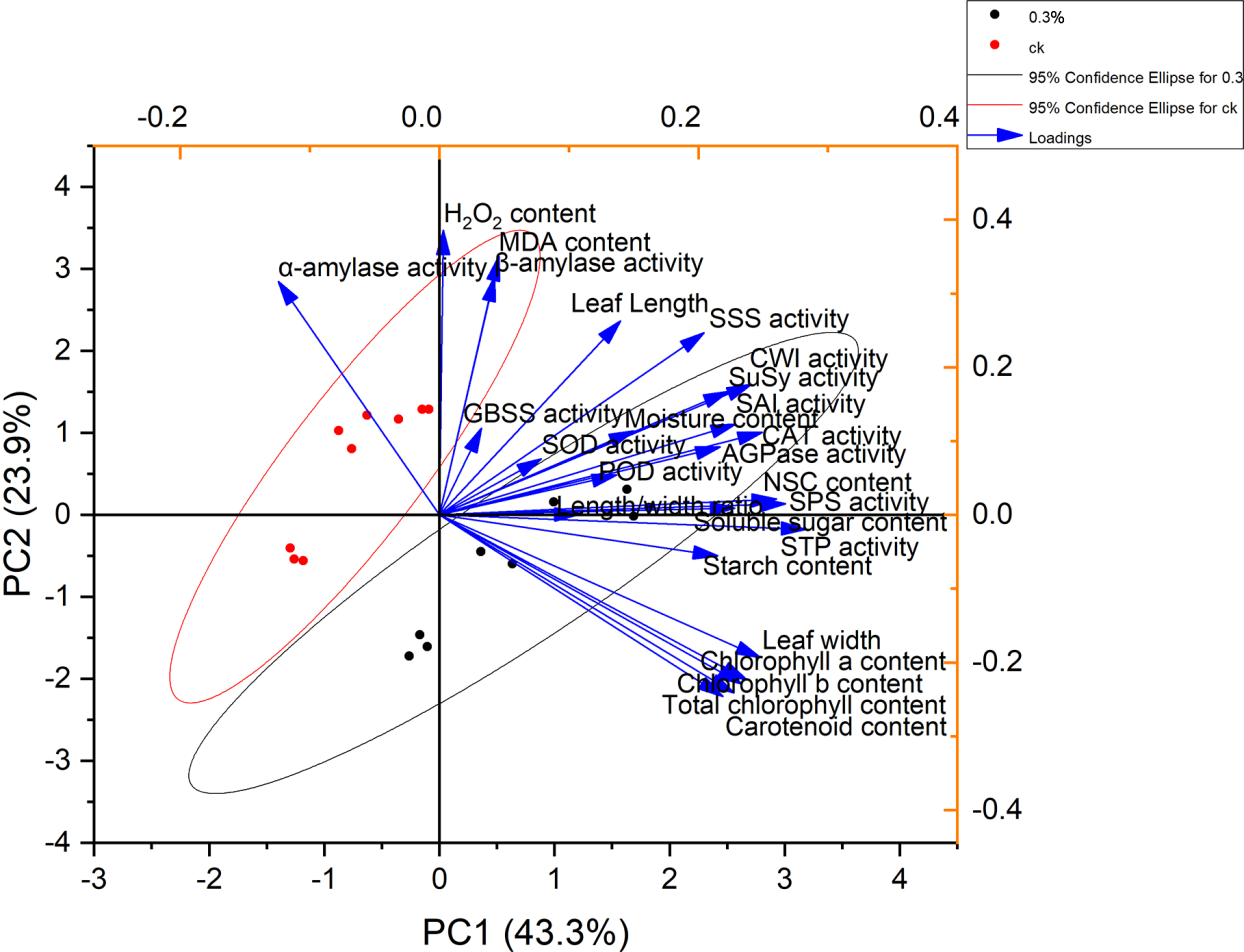
PCA analysis of leaf physiological parameters in *B. emeiensis* in response to foliar spraying of 0.3% urea. Black dots represent the 0.3% urea treatment group and red dots represent the control group. Ellipses indicate the 95% confidence intervals, and blue arrows represent the loading vectors.

In order to systematically evaluate the effect of nitrogen fertilizer on the physiological metabolic network of *B. emeiensis* leaves, principal component analysis (PCA) was performed on 23 key physiological and biochemical indexes (**Figure 7**). In this study, the cumulative variance contribution rate of the first two principal components (PC1 and PC2) reached 67.2%, of which PC1 explained 43.3% of the total variation and PC2 explained 23.9%, indicating that these two orthogonal dimensions can effectively capture the main information structure of the original data set. In the PCA score plot, the main representative variables of PC1 were NSC content, SPS activity, STP activity, while soluble sugar, starch and chlorophyll components also exhibited high loading values, indicating that PC1 primarily represented the accumulation of NSC and photosynthetic pigment levels in leaves. The main representative variables of PC2 were β-amylase activity, MDA content and H_2_O_2_ content, which reflected the characteristics related to starch degradation and oxidative stress responses. The PCA score plot showed significant spatial separation between the treatment group and the control group along the PC1 axis. Samples from the treatment group were mainly clustered in the positive region of PC1 and the negative region of PC2, while samples from the control group were distributed in the negative region of PC1 and the positive region of PC2. The aggregation of the treatment group in the positive direction of PC1 indicated that nitrogen fertilizer application significantly increased the photosynthetic pigment content of *B. emeiensis* leaves, enhanced the activities of SPS and STP, and promoted the transformation and accumulation of photosynthetic products into soluble sugars and non-structural carbohydrates.

## 4 Discussion

This study demonstrates that the application of nitrogen (particularly at 0.3%) during the branch extension and leaf extension phase significantly promote leaf growth and development in *B. emeiensis*. Moreover, this promotive effect is linked to enhanced environmental adaptability, achieved through improved sugar metabolism and maintained redox homeostasis.

In recent years, research on nutrient management and fertilization in bamboo plants has predominantly focused on soil-based fertilization techniques, whereas studies specifically addressing foliar fertilization in bamboo species remain limited, with relatively scarce available literature (**Table 2**). Most existing studies have emphasized the interactions between fertilization practices and soil nutrient status, while insufficient attention has been paid to how foliar nutrient supply directly influences bamboo growth, physiological responses, and developmental processes. Consequently, a systematic understanding of the application and effects of foliar fertilization in bamboo plants is still lacking (Cao et al., 2024; Fan et al., 2025; He et al., 2024; Huang et al., 2025; Januarie and Kadyampakeni, 2025; Li et al., 2025a; Liu et al., 2023; Tan et al., 2020; Yang et al., 2025; Zhao et al., 2025).

**Table 2:** Summary of research on bamboo nutrition and fertilization management over the past six years.

Number	Fertilizer	Bamboo species	Research objectives	Citation
1	Nitrogen fertilization	*Phyllostachys edulis*	Clarify how nitrogen fertilizer application affects the soil microbial community structure of bamboo forests through clonal integration.	Cao et al., 2024
2	Organic fertilizer	*Chimonobambusa opienensis*	Reveal the mechanism by which organic fertilizer combined with nitrogen-fixing bacteria (Azotobacter chroococcum) increases the yield of *Chimonobambusa opienensis* shoots through the “soil nutrient - microbial - metabolite” interaction network.	Fan et al., 2025
3	Biochar + N fertilizer	*Bambusa tuldoides ‘Swolleninternode’*	To study the effects of the combined application ratio of biochar and nitrogen fertilizer on the physiological characteristics of *Bambusa tuldoides* ‘Swolleninternode’ leaves and the physicochemical properties of the soil.	He et al., 2024
4	Sodium gluconate	*Dendrocalamus brandisii*	To evaluate the promoting effect of foliar spraying of sodium gluconate on the photosynthetic efficiency and the accumulation of photocontract compounds of *Dendrocalamus brandisi*i, as well as the differences in its responses at different vegetative growth stages.	Huang et al., 2025
5	Nitrogen	*Dendrocalamus asper*	To explore the dynamic response of nitrogen fertilizer application to the fertilization of annual dragon bamboo (*D. asper*) under the growth conditions in Florida (such as nutrient absorption, growth performance, etc.).	Januarie and Kadyampakeni, 2025
6	Potassium fertilizer	*Qiongzhuea tumidinoda*	To study the effects of potassium fertilizer application on the nutritional quality of *Qiongzhuea tumidinoda* shoots (such as amino acids, soluble sugars, etc.) and soil nutrient status.	Li et al., 2025a
7	Nitrogen,Si fertilization	*Phyllostachys edulis*	To evaluate the enhancing effect of different fertilization measures on the PhytOC sequestration capacity of *Phyllostachys edulis* under the background of temperature rise.	Liu et al., 2023
8	Nitrogen fertilizer	*Bambusa emeiensis*	To analyze the effects of different types of nitrogen fertilizers on the growth and development of *Bambusa emeiensis* shoots of Mount Emei during their growth period.	Tan et al., 2020
9	Sodium silicate	*Dendrocalamus brandisii*	To explore the effects of foliar spraying of sodium silicate on the concentration and morphological variation of phytophytes in the leaves of *Dendrocalamus brandisii*, as well as its responses at different vegetative growth stages.	Yang et al., 2025
10	Potassium fertilizer	*Neosinocalamus affinis*	To explore the effects of foliar application of potassium fertilizer on the anatomical structure and physiological characteristics of leaves of *Neosinocalamus affinis*	Zhao et al., 2025

Leaves are vital plant organs that accurately reflect a plant’s growth status, physiological changes, and environmental adaptability (Iqbal et al., 2025; Licaj et al., 2024; Poorter et al., 2009). Nitrogen significantly drives leaf development by promoting key physiological processes, such as chlorophyll synthesis and cell division (Li et al., 2025b, Li et al., 2021c; Qu et al., 2022; Tian et al., 2022b). In the present study, foliar nitrogen application significantly increased the length and width of *B. emeiensis* leaves, suggesting that nitrogen fertilization may enhance the leaf area index and thereby promote photosynthesis traits. Leaf expansion at the cellular level typically results from increased cell proliferation or enlargement (Jun et al., 2020). Microscopic observation of leaf anatomical structure in this study revealed continuous cell expansion during leaf development, with a trend toward larger cell sizes in the fertilized group compared to control (**Supplementary Table 1**; **Figure 2F**), indicating that foliar nitrogen application promotes leaf development through enhanced cellular expansion. These findings collectively suggest that the promotion of leaf area by foliar nitrogen is primarily driven by stimulating cellular expansion, a fundamental process underlying improved canopy development and photosynthetic potential.

Beyond promoting organ development, nitrogen fertilization also enhances leaf photosynthetic efficiency. Studies have shown that nitrogen application increases chlorophyll content and improves leaf photosynthetic capacity, particularly in newly developed leaves (Ahmad et al., 2021; Akkamis and Caliskan, 2024; Hikosaka, 2004). In *Bambusa tuldoides*, the combined application of nitrogen fertilizer and biochar can promote photosynthesis and leaf development (He et al., 2024). Furthermore, nitrogen promotes the accumulation of key photosynthetic intermediates in leaves, such as triose phosphates, sucrose, and pyruvate (Mawlong et al., 2023). In this study, we observed significant increment in the chlorophyll content in *B. emeiensis* following fertilizer application (**Figure 3**), which was accompanied by greater leaf length and width (**Figures 2B, C**). The increases in leaf dimensions and chlorophyll content are likely to enhance light capture capacity, developing the potential for higher photosynthetic rates and thereby promoting the synthesis of photosynthetic assimilates. These findings are consistent with the results reported by Noor et al. (2023) in wheat plants. This consistency suggests that these responses to nitrogen may represent a conserved physiological mechanism within the gramineous family.

Following foliar nitrogen application, the contents of soluble sugars and starch in the new leaves of *B. emeiensis* increased significantly (**Figures 4A, B**), which may be attributed to corresponding changes in the activities of sucrose phosphate synthase (SPS) and key starch-synthesizing enzymes, such as AGPase, GBSS, and SSS (**Figures 5A, E–G**). These results suggest that foliar urea application may promote the accumulation of photosynthetic carbohydrates in leaves by enhancing the activities of these enzymes and improving photosynthetic carbon assimilation capacity. Similar findings were reported by Amoah et al. (2025), who observed that nitrogen application significantly promoted shoot and root development, biomass accumulation, and overall phenotypic performance in maize, along with enhanced photosynthetic activity, and increased sugar and starch accumulation. Mu et al. (2024) also demonstrated that nitrogen fertilization facilitated the accumulation of photosynthetic carbohydrates in plant leaves. This indicates that foliar nitrogen orchestrates a coordinated upregulation of both carbon fixation and storage metabolism.

Enzymes, such as SAI, CWI, SuSy, and SPS play crucial roles in sucrose metabolism and are associated with plant growth, development, and stress responses (Khan et al., 2024; Li et al., 2025a., Li et al., 2025b; Wang et al., 2019b; Zhang et al, 2019). The increased SPS activity following nitrogen application (**Figure 5A**) indicates that the accumulation of soluble sugars may result from enhanced sucrose synthesis. Concurrently, the elevated activities of sucrose-degrading enzymes, i.e., CWI, SAI, and SuSy (**Figures 5B–D**) suggest that more sucrose is being decomposed to support leaf development, which aligns with the observed lower soluble sugar content mentioned earlier (**Figure 4A**). Among starch metabolic enzymes, AGPase, GBSS, and SSS are key catalysts in the starch synthesis pathway, whereas STP plays a major role in starch degradation (Ballicora et al., 2004; Dauvillee et al., 2006; Pfister and Zeeman, 2016; Qu et al., 2018; Schupp and Ziegler, 2004; Zhang et al., 2016). Research has demonstrated that nitrogen application in rice (*Oryza sativa*) can enhance the activities of AGPase and GBSS enzymes, thereby improving grain transparency (Fan et al., 2024b). Zhao et al. (2025) found that the application of foliar potassium to *Neosinocalamus affinis* can modulate the activity of the enzymes SuSy, AGPase, and SSS during the conversion of sucrose to starch, thus enhancing starch accumulation. In the present study, foliar nitrogen application similarly increased the activities of the aforementioned starch synthesis-related enzymes, a result consistent with the observed trend of increased starch content.

A mineral nutrient influences plant resilience to environmental stresses (Hossain et al., 2024; Reis et al., 2015). Appropriate nitrogen fertilization can enhance the activities of antioxidant enzymes in plants, reduce the accumulation of ROS under stress conditions, and thereby protect cell membranes and organelles from oxidative damage (Ahanger and Ahmad, 2019). In maize (*Zea mays*), urea application increased antioxidant enzyme activities and delayed leaf senescence process (Ahmad et al., 2021). Nitrogen application in *Fargesia denudata* can enhance drought tolerance by boosting the activity of antioxidant enzymes under drought-resistant conditions (Wu et al., 2022). In this study, the urea application significantly downregulated MDA and H^2^O^2^ levels in newly emerged leaves of *B. emeiensis*. Measurements of antioxidant-related enzyme activities revealed increased SOD, CAT, and POD activities, demonstrating that nitrogen fertilization alleviates oxidative stress in plants by enhancing the capacity of the antioxidant system.

## 5 Conclusion and future research directions

**Figure 8:**
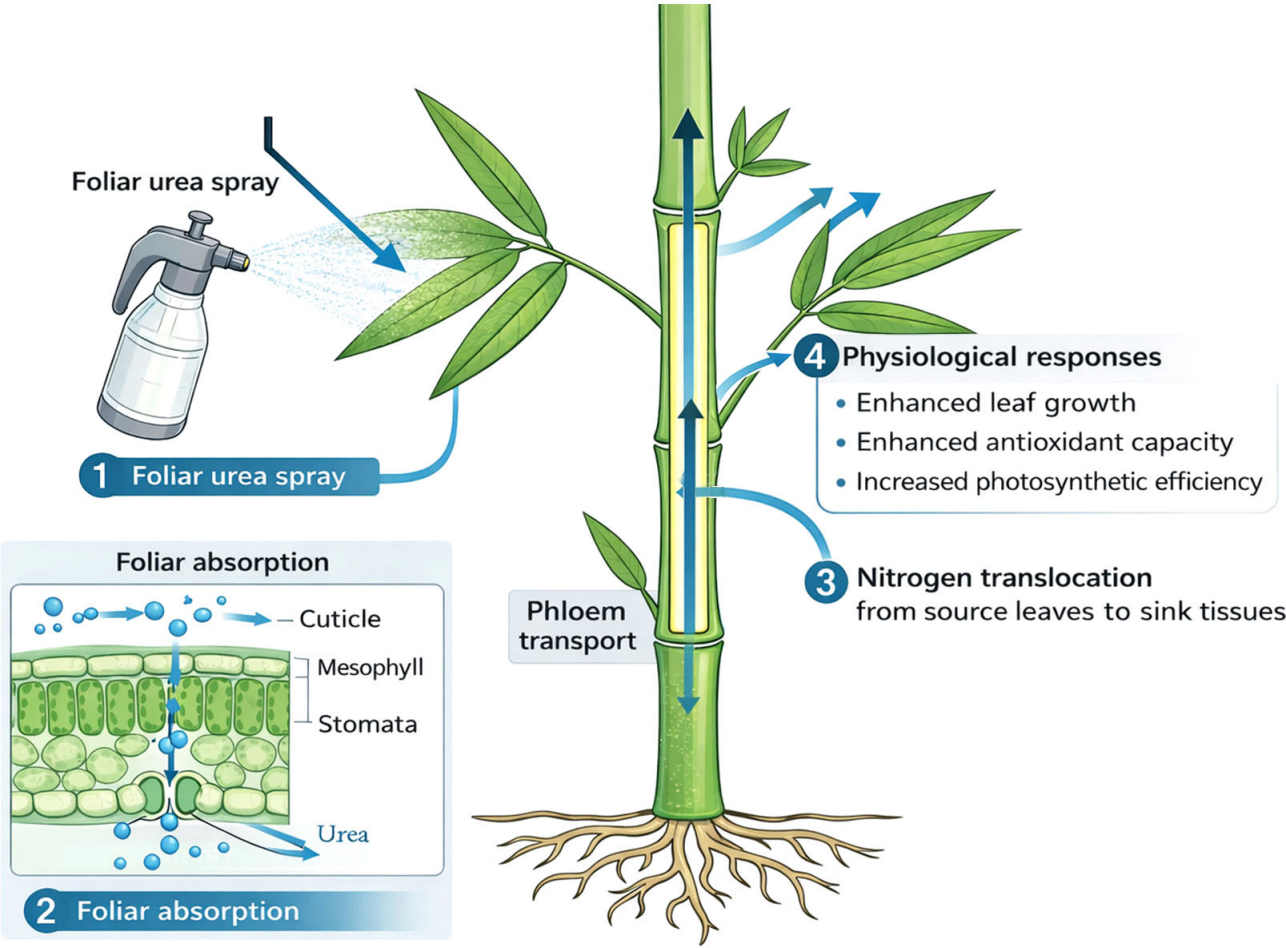
Schematic diagram summarizing the promotion of leaf growth and stress resistance by foliar urea in *B. emeiensis*.

This study revealed that foliar urea application significantly increased leaf length, width, and photosynthetic pigments in newly emerged leaves of *B. emeiensis* (**Figures 2**, **3**). Further analysis revealed enhanced levels of soluble sugars and starch, likely due to increased activity of key metabolic enzymes (**Figures 4**, **5A-J**). Additionally, it enhanced the activities of crucial antioxidant enzymes (SOD, CAT, and POD) and reduced oxidative metabolite accumulation, potentially improving environmental adaptability (**Figure 5K-O**). In summary, foliar application of nitrogen (0.3% urea during the branch and leaf extension phase, ~May) coordinately promoted leaf growth, photosynthetic assimilation, and antioxidant capacity (**Figure 8**). While this study delineates these positive physiological responses, we acknowledge that a more complete assessment of agronomic efficiency and nitrogen use economy would be strengthened by future measurements of biomass production (e.g., specific leaf weight) and leaf nitrogen content per unit area. These parameters would allow for the direct calculation of photosynthetic nitrogen-use efficiency and yield-level nitrogen productivity, crucial for a full cost-benefit analysis.

These findings provide a scientific basis for a precise and potentially cost-effective management strategy. The defined optimal dose and timing enable efficient nutrient use, minimizing waste. This approach is particularly suited for scalable applications such as drone-assisted spraying, which can overcome terrain challenges, reduce labor costs, and promote sustainable, high-yield bamboo cultivation. Future research will focus on examining how varying different nitrogen forms (urea, ammonium nitrate, amino acid-based) and concentrations to identify optimal dose–response relationships for newly emerged leaves. Determine the most effective phenological stage (s) for foliar spraying (e.g., leaf expansion, pre-photosynthetic activation) and evaluate single *vs*. split applications. Exploring the interaction between foliar nitrogen treatments and soil nutrient profiles to identify synergistic effects on overall plant health. Incorporating biomass and nitrogen content metrics to fully quantify the economic and physiological efficiency of this practice.

## Data Availability

The original contributions presented in the study are included in the article/**Supplementary Material**. Further inquiries can be directed to the corresponding authors.
