# Supplementary material for: Effects of foliar nitrogen application on morphological and physiological characteristics in newly emerged leaves of Bambusa emeiensis
Source: Front Plant Sci. 2026 Mar 30;17:1751417. doi: 10.3389/fpls.2026.1751417 (PMC13070964; doi:10.3389/fpls.2026.1751417)
Supplement: Supplementary file 2 [file DataSheet2.docx]

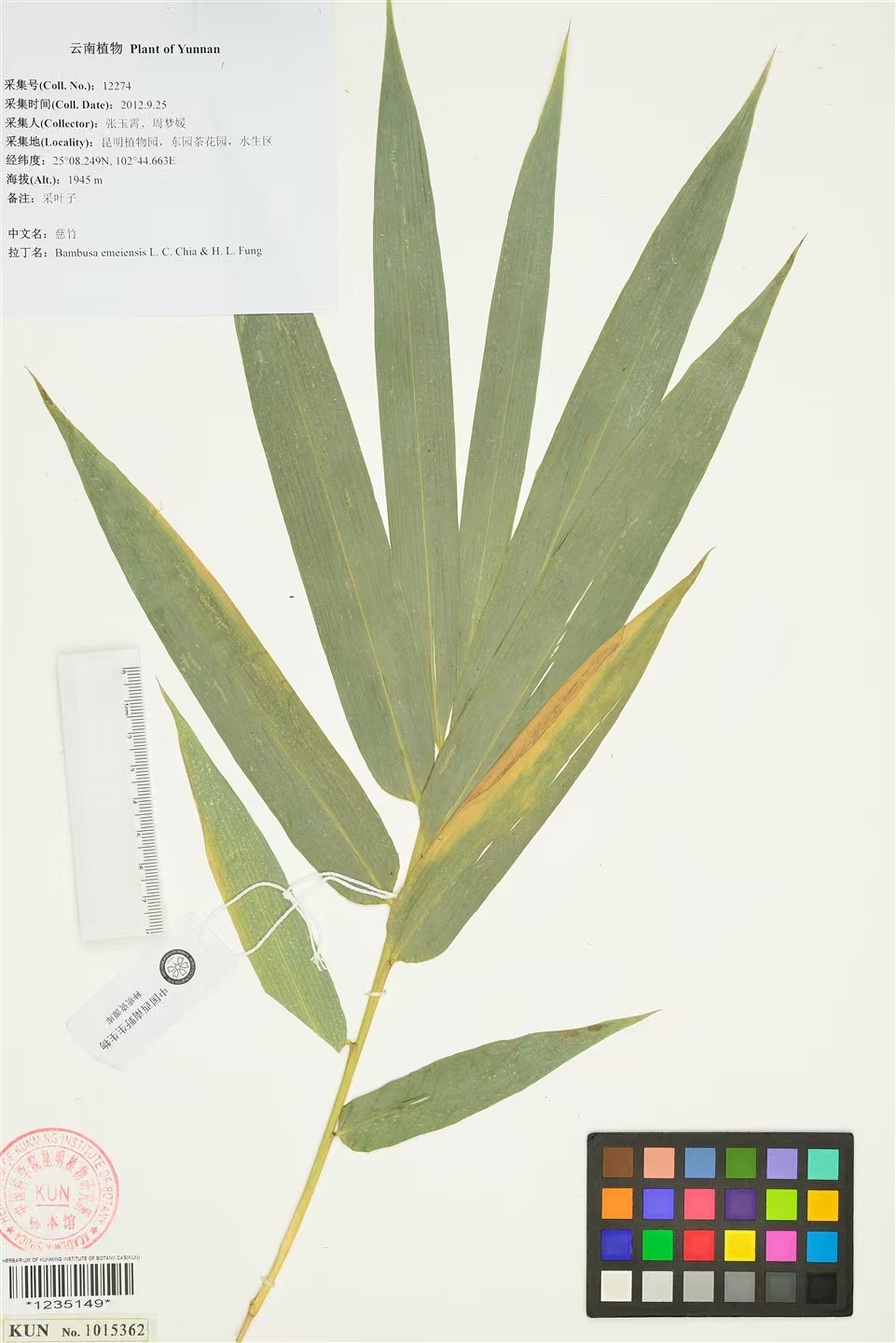


Fig. S1. Supplementary Figure S1. Herbarium specimen of *Bambusa emeiensis* L.C. Chia & H.L. Fung (KUN No. 1015362) deposited at the Herbarium of Kunming Institute of Botany, Chinese Academy of Sciences (KUN). Image accessed via the Chinese Virtual Herbarium (CVH) for academic and non-commercial purposes.


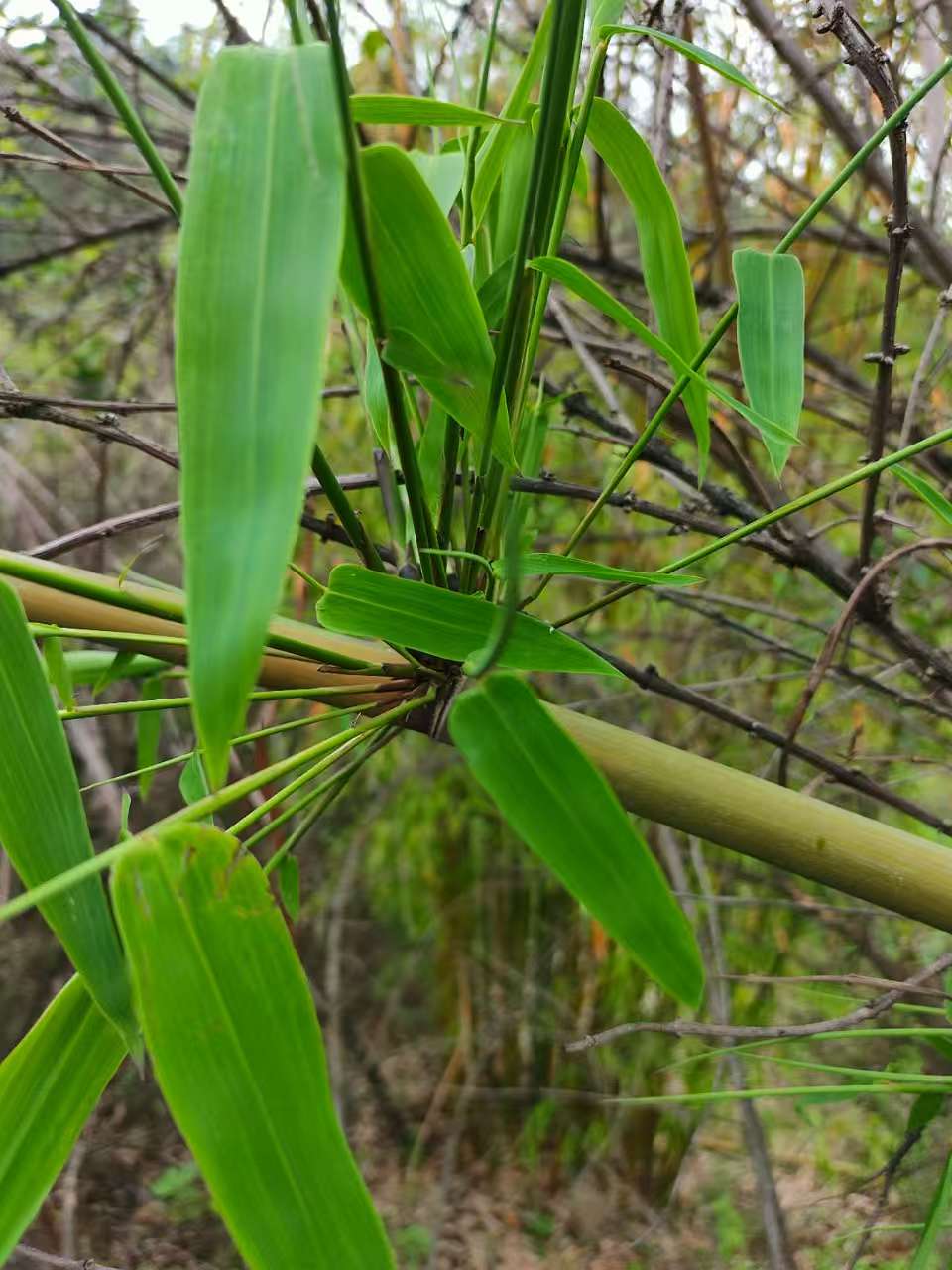


Fig. S2. New leaves of *B. emeiensis* in May. Scale bar = 2.5 cm.


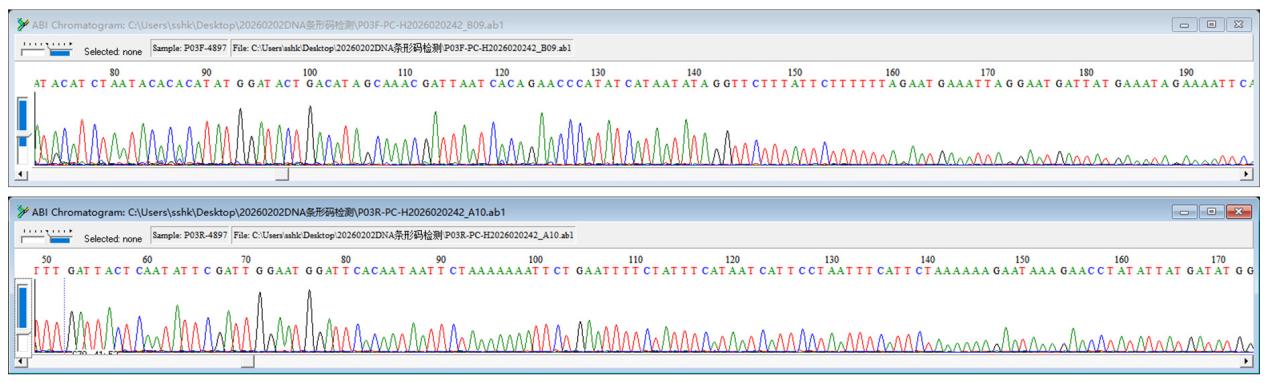


Fig. S3. Representative sequencing chromatogram of the trnL gene-specific PCR product amplified from *B. emeiensis* leaf.


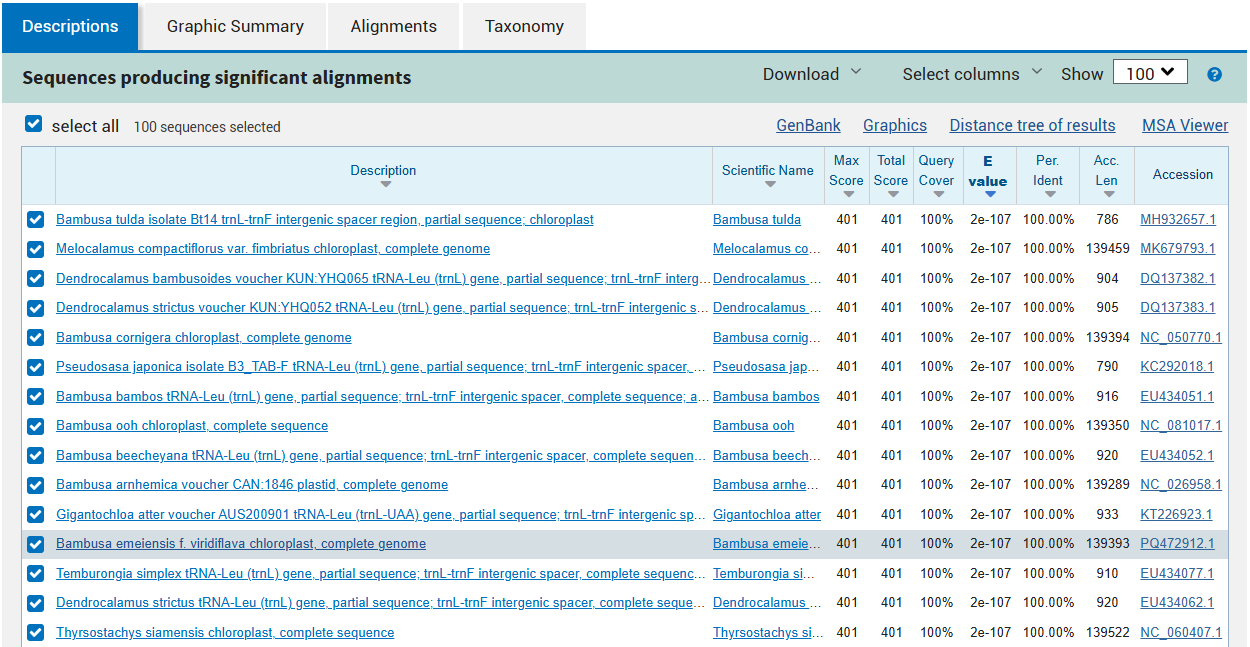


Fig. S4. BLASTn comparison of the trnL gene sequence from *B. emeiensis* leaf against reference sequences in the NCBI database.


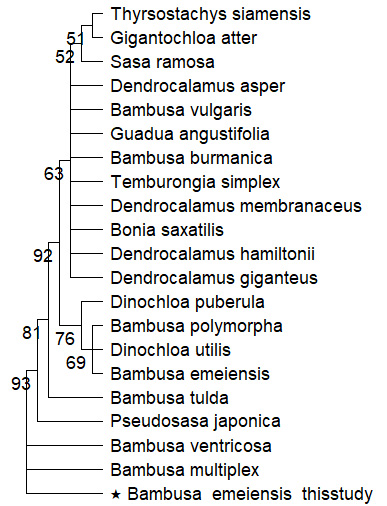


Fig. S5**.** Phylogenetic tree based on the chloroplast trnL (UAA) intron sequences of *B. emeiensis* and related bamboo species retrieved from the NCBI database. The sequences of the top BLAST hits (15–20 taxa) were included for comparison. The phylogenetic tree was constructed using the Neighbor-Joining method, and bootstrap values (%) based on 1000 replicates are shown at the nodes. The sequence generated in this study is marked with a star (★).


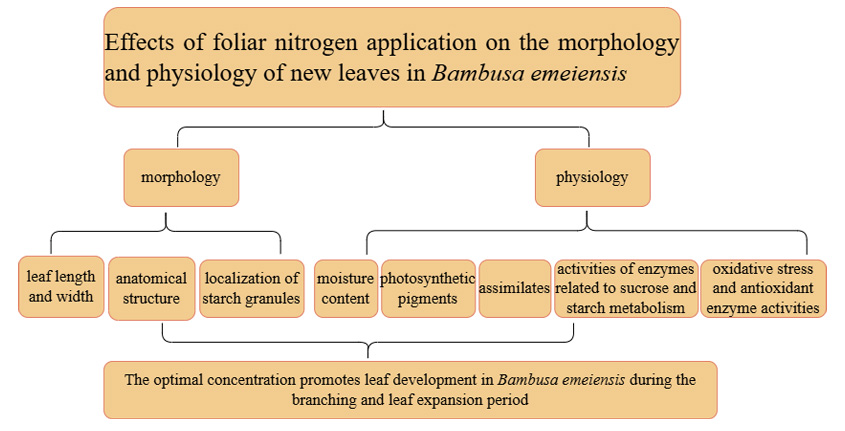


Fig. S6. Method process

Supplementary Table 1. Effects of Foliar 0.3% CH_4_N_2_O Application at on the Anatomical Structure of New Leaves in *B. emeiensis*

| **Morphological Index** | **Treatment** | | | | | | |
| --- | --- | --- | --- | --- | --- | --- | --- |
|  | **Control** | | | **0.3%CH_4_N_2_O** | | | |
|  | **7D** | **14D** | **21D** | | **7D** | **14D** | **21D** |
| **Leaf Thickness** | **133.59±6.46b** | **140.8±11.08bc** | **142.92±15.18d** | | **132.61±13.23b** | **144.92±7.88ab** | **178.57±10.74a** |
| **Thickness of Mesophyll Cell(μm)** | **105.4±27.09ab** | **105.46±19.17b** | **105.95±12.13c** | | **105.46±10.12ab** | **108.02±11.05b** | **128.59±13.74a** |
| **Thickness of Adaxial Epidermal Cell(μm)** | **19.73±4.88b** | **23.1±2.82a** | **24.67±4.15b** | | **21.55±3.64a** | **23.75±4.67a** | **30.44±3.91a** |
| **Thickness of Abaxial Epidermal Cell(μm)** | **15.82±1.99bc** | **16.01±2.85ab** | **16.08±2.71c** | | **16.38±3.59ab** | **16.83±2.49ab** | **21.14±3.84a** |
| **Bulliform Cell Area** | **1746.52±286.68a** | **1818.04±700.37ab** | **1952.24±558.65cd** | | **1963.49±759.26a** | **2058.76±574.59a** | **3171.63±557.55a** |

Different letters with the same growth period indicated signiﬁcant differences at *p* < 0.05 according to the Duncan’s test. Data are expressed as mean ± standard deviation (SD).
